# Three-Dimensional Imaging of the Intracellular Fate of Plasmid DNA and Transgene Expression: ZsGreen1 and Tissue Clearing Method CUBIC Are an Optimal Combination for Multicolor Deep Imaging in Murine Tissues

**DOI:** 10.1371/journal.pone.0148233

**Published:** 2016-01-29

**Authors:** Shintaro Fumoto, Koyo Nishimura, Koyo Nishida, Shigeru Kawakami

**Affiliations:** Graduate School of Biomedical Sciences, Nagasaki University, Nagasaki, Japan; Medical College of Georgia, UNITED STATES

## Abstract

Evaluation methods for determining the distribution of transgene expression in the body and the *in vivo* fate of viral and non-viral vectors are necessary for successful development of *in vivo* gene delivery systems. Here, we evaluated the spatial distribution of transgene expression using tissue clearing methods. After hydrodynamic injection of plasmid DNA into mice, whole tissues were subjected to tissue clearing. Tissue clearing followed by confocal laser scanning microscopy enabled evaluation of the three-dimensional distribution of transgene expression without preparation of tissue sections. Among the tested clearing methods (Clear^T2^, SeeDB, and CUBIC), CUBIC was the most suitable method for determining the spatial distribution of transgene expression in not only the liver but also other tissues such as the kidney and lung. In terms of the type of fluorescent protein, the observable depth for green fluorescent protein ZsGreen1 was slightly greater than that for red fluorescent protein tdTomato. We observed a depth of ~1.5 mm for the liver and 500 μm for other tissues without preparation of tissue sections. Furthermore, we succeeded in multicolor deep imaging of the intracellular fate of plasmid DNA in the murine liver. Thus, tissue clearing would be a powerful approach for determining the spatial distribution of plasmid DNA and transgene expression in various murine tissues.

## Introduction

The development of gene delivery systems requires methods to monitor transgene expression. Firefly luciferase is often employed as a reporter gene. It has high sensitivity and a relatively short half-life [1]. Thus, firefly luciferase is suitable for quantitative analysis of transgene expression. Fluorescent proteins, such as enhanced green fluorescent protein and Discosoma red fluorescent protein [2], can visualize transgene expression and are suitable for determining the distribution, number and type of transgene-positive cells [3]. In general, to observe fluorescent proteins in tissues, it is necessary to prepare thin tissue sections because of the absorbance of excitation light, scattering, and autofluorescence. Delicate techniques are required to prepare fine sections of tissues. Reconstruction of a three-dimensional image after tissue sectioning is difficult because it is easy to lose the specimen during consecutive tissue sectioning. Two-photon excitation microscopy [4] can overcome this problem. It can observe a depth of ~700 μm within intact tissues. However, multicolor imaging by two-photon excitation microscopy is relatively difficult compared with confocal laser scanning microscopy (CLSM), because two-photon excitation wavelengths are highly dependent on fluorochromes and often overlap with each other [5].

In addition to transgene expression, the biodistribution of vectors is an important issue. The use of radioisotopes has enabled quantification of the amount of vector in each tissue [6–8]. Moreover, the sensitivity of radioisotopes is extremely high compared with fluorescent probes. In contrast, fluorescence has several advantages to determine the cellular distribution and intracellular fate of the vector because of multicolor staining. However, it is also necessary to prepare thin tissue sections for observation of the vector.

Here, we applied tissue clearing methods [9] to observe fluorescence in whole tissue specimens without the need to prepare tissue sections. Several tissue clearing methods have been developed, especially for the brain in neuroscience [10–16]. Among the fluorescent protein-compatible methods, we applied Clear^T2^ [10], SeeDB [11], and CUBIC [12, 13] because of their simplicity (i.e. immersion in clearing solutions). In this study, we compared these methods to determine the optimal approach to observe three-dimensional transgene expression in tissues. Among the variety of fluorescent proteins, we chose bright fluorescent proteins ZsGreen1 [17] and tdTomato [18, 19] as green and red fluorescent proteins, respectively, to compare their effectiveness for deep imaging. We simultaneously delivered two plasmid DNAs encoding ZsGreen1 and tdTomato by hydrodynamic injection into mice. To test the applicability of the tissue clearing method, transgene expression was evaluated in not only the liver, but also other tissues including the heart, kidney, spleen, lung, stomach, and small and large intestines. Moreover, we applied the method to elucidate the *in vivo* intracellular fate of plasmid DNA in the liver after hydrodynamic injection.

## Materials and Methods

### Reagents

Paraformaldehyde, formamide, fructose, urea, 2,2′,2″-nitrilotriethanol, and sucrose were purchased from Wako Pure Chemical Industries, Ltd. (Osaka, Japan). Polyethyleneglycol (PEG; molecular weight: 4,000) and Triton X-100 were purchased from Nacalai Tesque (Kyoto, Japan). *N*,*N*,*N′*,*N′*-tetrakis(2-hydroxypropyl)ethylenediamine and α-thioglycerol were purchased from Tokyo Chemical Industry Co., Ltd. (Tokyo, Japan). 4′,6-Diamidino-2-phenylindole dihydrochloride (DAPI) was purchased from Sigma-Aldrich Co. Llc. (St. Louis, MO, USA). Tetramethylrhodamine (TMR)-labeled dextran (molecular weight: 10,000; lysine fixable) was purchased from Molecular Probes, Inc. (Eugene, OR, USA).

### Plasmid DNA

The pCMV-luciferase vector encoding firefly luciferase under the control of the cytomegalovirus promoter was constructed previously [20]. Plasmid DNAs pZsGreen1-N1 encoding green fluorescent protein ZsGreen1 and ptdTomato-C1 encoding red fluorescent protein tdTomato were purchased from Clontech (Takara Bio inc., Shiga, Japan). Plasmid DNA was amplified in *Escherichia coli* strain DH5α, isolated, and purified using an EndoFree® Plasmid Giga Kit (Qiagen GmbH, Hilden, Germany). Plasmid DNA was stored at −20°C prior to experiments. TMR- or Cy5-labeled plasmid DNAs were prepared using a Label IT Nucleic Acid Labeling Kit (Mirus Co., Madison, WI, USA).

### Animals

Male ddY mice (5 weeks old, 25.5–28.5 g) were purchased from Kyudo Co., Ltd. (Kumamoto, Japan). They were housed in an air-conditioned room and maintained on a standard laboratory diet (MF, Oriental Yeast, Co., Ltd., Tokyo, Japan) and water *ad libitum*. All animal experiments were carried out in accordance with the Guide for the Care and Use of Laboratory Animals, as adopted and promulgated by the U.S. National Institutes of Health, and the Guidelines for Animal Experimentation of Nagasaki University. The protocol was approved by the Animal Care and Use Committee of the Nagasaki University (Permission number: 1304171055). Surgeries were performed under sodium pentobarbital anesthesia, and all efforts were made to minimize suffering.

### *In vivo* gene transfer

A high-volume solution of plasmid DNAs (10 μg/2.5 mL in saline) was rapidly injected (within 5 s) into the tail vein of mice. In some experiments, TMR-dextran (250 μg/mL) was co-injected with plasmid DNA. At 24 h after injection, the mice were subjected to luciferase assay or perfusion fixation under anesthesia with pentobarbital. For perfusion, a 26 G needle connected to a tube was passed through the left ventricle. The perfusates were 10 mL of phosphate-buffered saline (PBS) containing 10 U/mL heparin, 50 mL PBS, 200 mL of 4% paraformaldehyde in PBS, and 50 mL PBS in this order. After perfusion fixation, tissue samples were collected from mice and subjected to tissue clearing.

### Tissue clearing

Tissue clearing was performed according to the original methods reported elsewhere [10–13]. Depending on the method, fixed tissues were immersed in a tissue clearing solution(s) for several days. For Clear^T2^, immersion times were 1, 1 and 24 h for 25% formamide/10% PEG, 50% formamide/20% PEG, and 50% formamide/20% PEG, respectively. For SeeDB, immersion times were 4–8, 4–8, 4–8, 12, 12 and 24 h for 20%, 40%, 60%, 80%, 100% and 115% (weight per volume) fructose with 0.5% α-thioglycerol, respectively. For CUBIC, fixed tissues were immersed in reagent 1 (25% *N*,*N*,*N′*,*N′*-tetrakis(2-hydroxypropyl)ethylenediamine, 25% urea, and 15% Triton X-100) for 5 days (the solution was changed every day), soaked in PBS for 1 day, and then immersed in reagent 2 (10% 2,2′,2″-nitrilotriethanol, 25% urea, 50% sucrose, and 0.1% Triton X-100) for 4 days. For nuclear staining, 5 μg/mL DAPI was added to each reagent. As a control, tissues were immersed in PBS for 1 day.

### CLSM

A whole cleared tissue was mounted on a glass-based dish (No.1S, AGC Techno Glass Co., Ltd., Shizuoka, Japan) and subjected to CLSM. Z-stack images were acquired under an inverted confocal microscope (LSM 710 with spectral imaging equipment, 405, 488, 543 and 633 nm laser lines, Carl Zeiss Microimaging GmbH, Jena, Germany). The acquisition software was ZEN2008. Objective lenses were a 20× dry lens (EC Plan-Neofluar, numerical aperture (NA): 0.5; working distance (WD): 2.0 mm), 40× oil-immersion lens (EC Plan-Neofluar, NA: 1.30; WD: 0.21 mm), long distance 40× water-immersion lens (C Apochromat, NA: 1.10; WD: 0.62 mm), and 63× oil-immersion lens (Plan-Apochromat, NA: 1.4; WD: 0.19 mm). Z-intervals for these lenses were set to 2.118, 0.600, 0.800, and 0.366 μm, respectively.

### Luciferase assay

To detect firefly luciferase activities, the tissues were washed twice with saline and homogenized in lysis buffer consisting of 0.1 M Tris-HCl (pH 7.8) containing 0.05% Triton X-100 and 2 mM EDTA. The volume of lysis buffer added was 4 μL/mg of tissue. The homogenates were centrifuged at 15,000 × *g* for 5 min. Twenty microliters of supernatant were then mixed with 100 μL of luciferase assay substrates (PicaGene®, Toyo Ink Mfg. Co., Ltd., Tokyo). The light produced was measured immediately using a luminometer (MiniLumat LB 9506; Berthold Technologies, Bad Wildbad, Germany). Luciferase activities were indicated as the relative light units (RLU) per gram of tissue.

### Photostability assay

To check the photostabilities of ZsGreen1 and tdTomato, the tissues were washed twice with saline and homogenized in PBS (4 μL/mg of tissue). The homogenates were centrifuged at 15,000 × *g* for 5 min, and then the supernatants were mixed with equal volumes of 4% paraformaldehyde in PBS. At 2 hours after fixation, residual paraformaldehyde was removed by passing through a PD-10 desalting column (GE Healthcare Bio-sciences Corp, Piscataway, NJ, USA). Fifty microliters of the sample were mixed with 2 mL PBS or CUBIC reagent 2. Changes in the fluorescent intensities of ZsGreen1 (emission 510 nm) and tdTomato (emission 570 nm) in PBS or CUBIC reagent 2 by consecutive irradiation of the excitation light (488 or 543 nm, respectively) were monitored using a spectrofluorophotometer (RF-1500, Shimadzu Corp., Kyoto, Japan).

## Results

### Comparison of clearing methods

**Fig 1:**
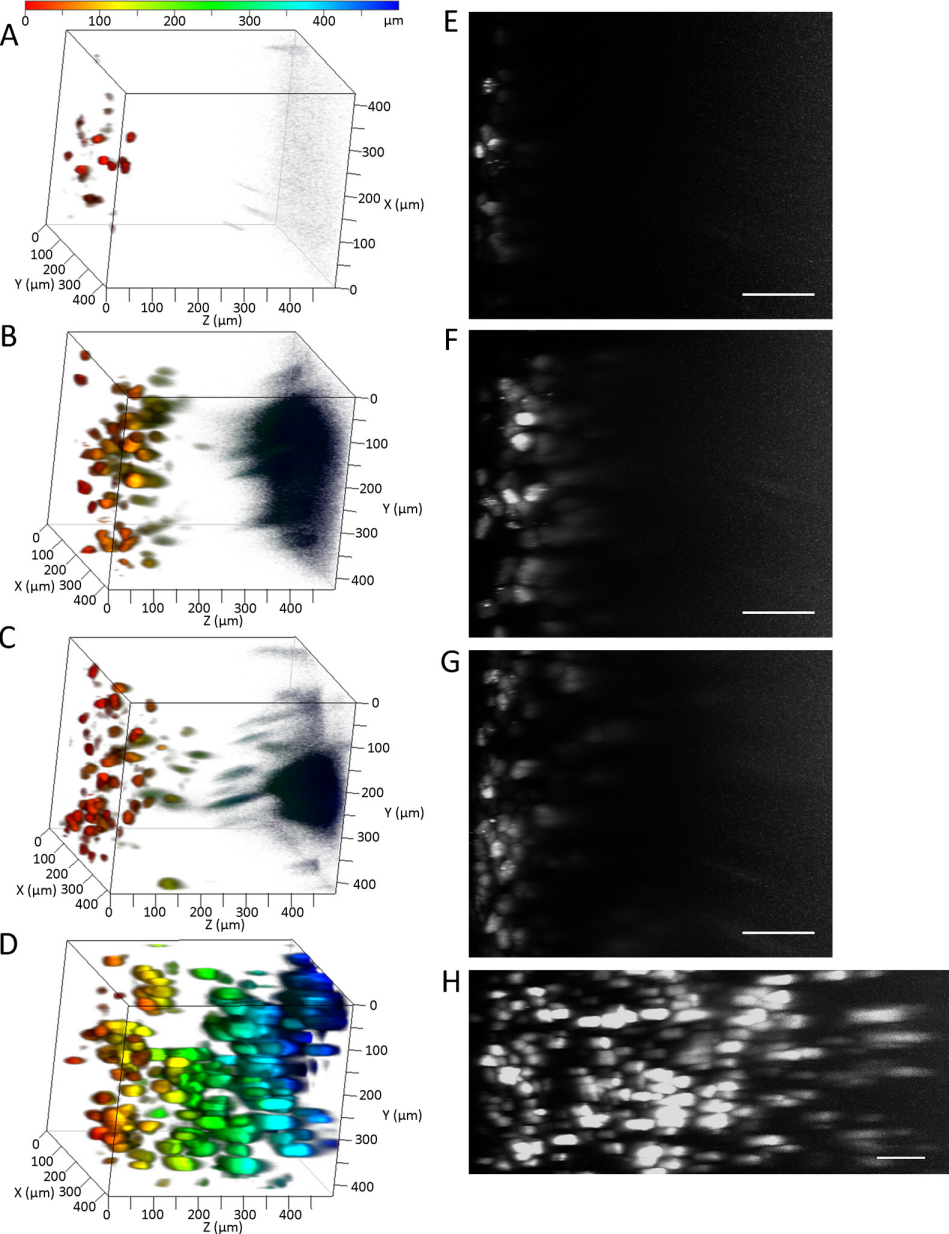
Comparison of tissue clearing methods. (A, E) PBS, (B, F) Clear^T2^, (C, G) SeeDB, and (D, H) CUBIC. ZsGreen1 expression in the liver was detected by CLSM. (A–D) Depth coding. The color chart indicates depth on the Z-axis. (E–H) Maximum intensity projection (X–Z plane). Scale bar: 100 μm. Acquisition conditions: lens, 20× dry; laser, 488 nm; output, 5%; emission, 492–540 nm; master gain, (A, E) 432–1200, (B, F) 433–1129, (C, G) 405–1129, and (D, H) 492–1115.

**Fig 2:**
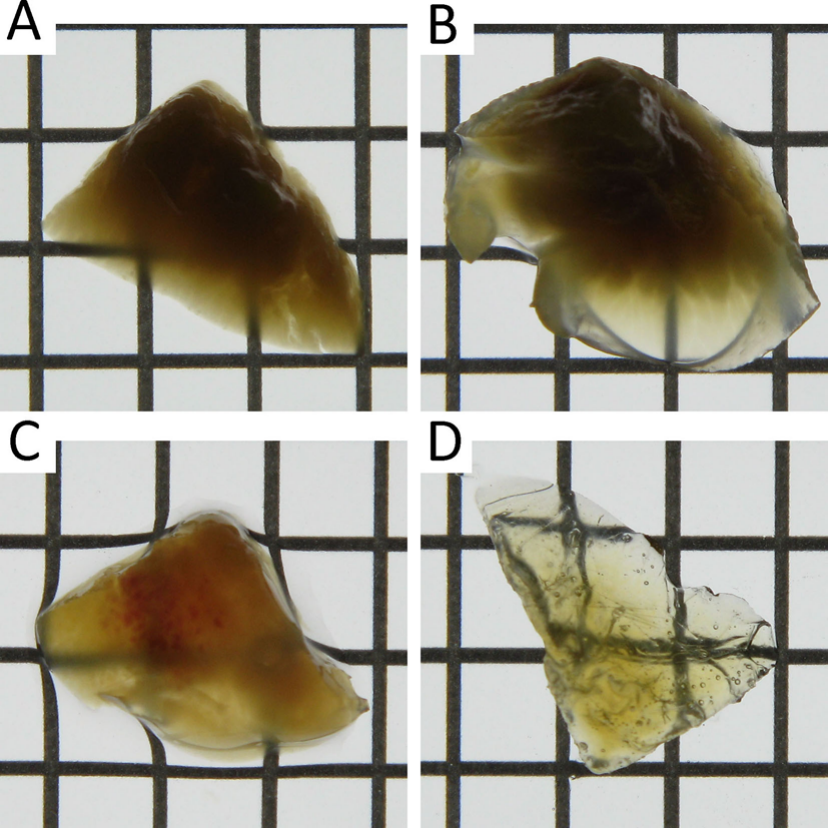
Transmission color images of the liver after tissue clearing. (A) PBS, (B) Clear^T2^, (C) SeeDB, and (D) CUBIC. Each lattice indicates 4×4 mm.

To determine an optimal method for three-dimensional imaging of whole tissue, we compared the observable depths using Clear^T2^, SeeDB, and CUBIC protocols. Because transgene expression in the liver is the highest among all tissues after hydrodynamic injection, the liver was chosen for comparisons. To increase the sensitivity in deep regions, the master gain of the photomultiplier tube in CLSM was adjusted linearly. Among the clearing methods, CUBIC was optimal for deep imaging of ZsGreen1 transgene expression in the liver (**Fig 1**). The results of CLSM were consistent with those of transmitted images of the liver (i.e. the liver immersed in CUBIC reagents was the most transparent) (**Fig 2**). Because the master gains were near maximal at a depth of 500 μm using PBS, Clear^T2^, and SeeDB, we observed extensive noise in deep regions. In contrast, the master gains using CUBIC at depths of 500 μm and 1 mm were about 67% and 93%, respectively. The observable depths without sectioning the liver were approximately 70, 200, 240, and 1000 μm using PBS, Clear^T2^, SeeDB, and CUBIC, respectively. Prolonging the immersion times of Clear^T2^ and SeeDB did not improve transparencies (**Figure A in S1 File**) or observable depths (**Figure B in S1 File**).

### Comparison of fluorescent proteins

**Fig 3:**
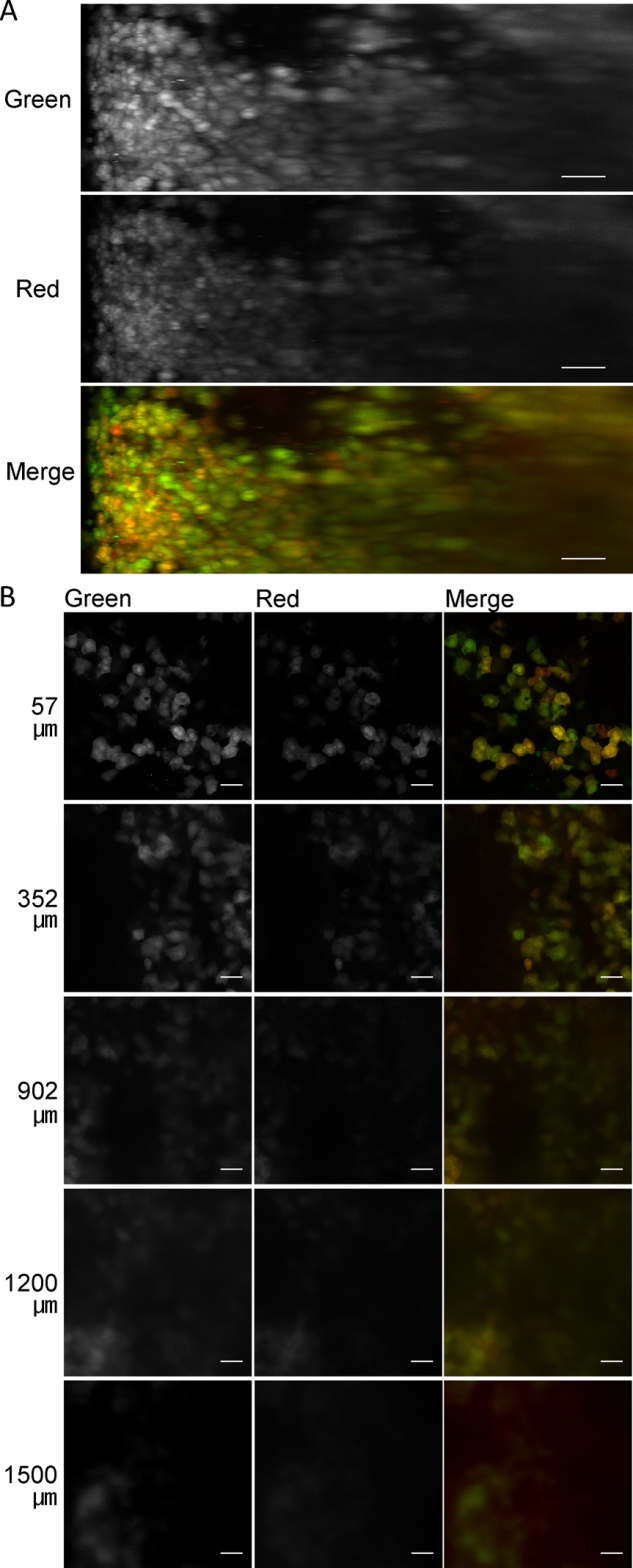
Comparison of the observable depth with fluorescent proteins ZsGreen1 (green) and tdTomato (red) in the liver treated with CUBIC. (A) Maximum intensity projections (X–Z plane). Scale bar: 100 μm. (B) Single plane images at the indicated depth (μm) from the liver surface. Scale bar: 50 μm. Acquisition conditions: lens, 20× dry; laser, 488 nm (output, 1%–12%; emission, 492–540 nm; master gain, 480–766) and 543 nm (output, 5%–100%; emission, 550–670 nm; master gain, 600–906).

Because autofluorescence and absorption depend on the wavelength of light, we tested the performance of two bright fluorescent proteins, ZsGreen1 and tdTomato. Because the output of the 543 nm laser (1 mW) was much lower than that of the 488 nm laser (30 mW), not only the master gains but also the laser outputs were linearly adjusted in the next experiment (**Fig 3**). After simultaneous hydrodynamic injection of two plasmid DNAs encoding ZsGreen1 and tdTomato, both ZsGreen1 and tdTomato were successfully visualized in deep regions (**Fig 3A**) and mostly expressed in the same cells (**Fig 3B**). Increasing the laser output improved the observable depth of ZsGreen1 (1500 μm). The observable depth of tdTomato (1200 μm) was slightly lower than that of ZsGreen1. In addition, ZsGreen1 was more photostable than tdTomato in CUBIC reagent 2 (**Figure C in S1 File**). When plasmid DNA encoding tdTomato was hydrodynamically injected into mice, a small amount of fluorescence was observed in the green channel with 488 nm laser excitation (**Figure D in S1 File**). Thus, ZsGreen1 was more suitable for deep imaging than tdTomato. Subsequently, we used ZsGreen1 as the transgene for the following experiments.

### Three-dimensional observations of transgene expression in various tissues

**Fig 4:**
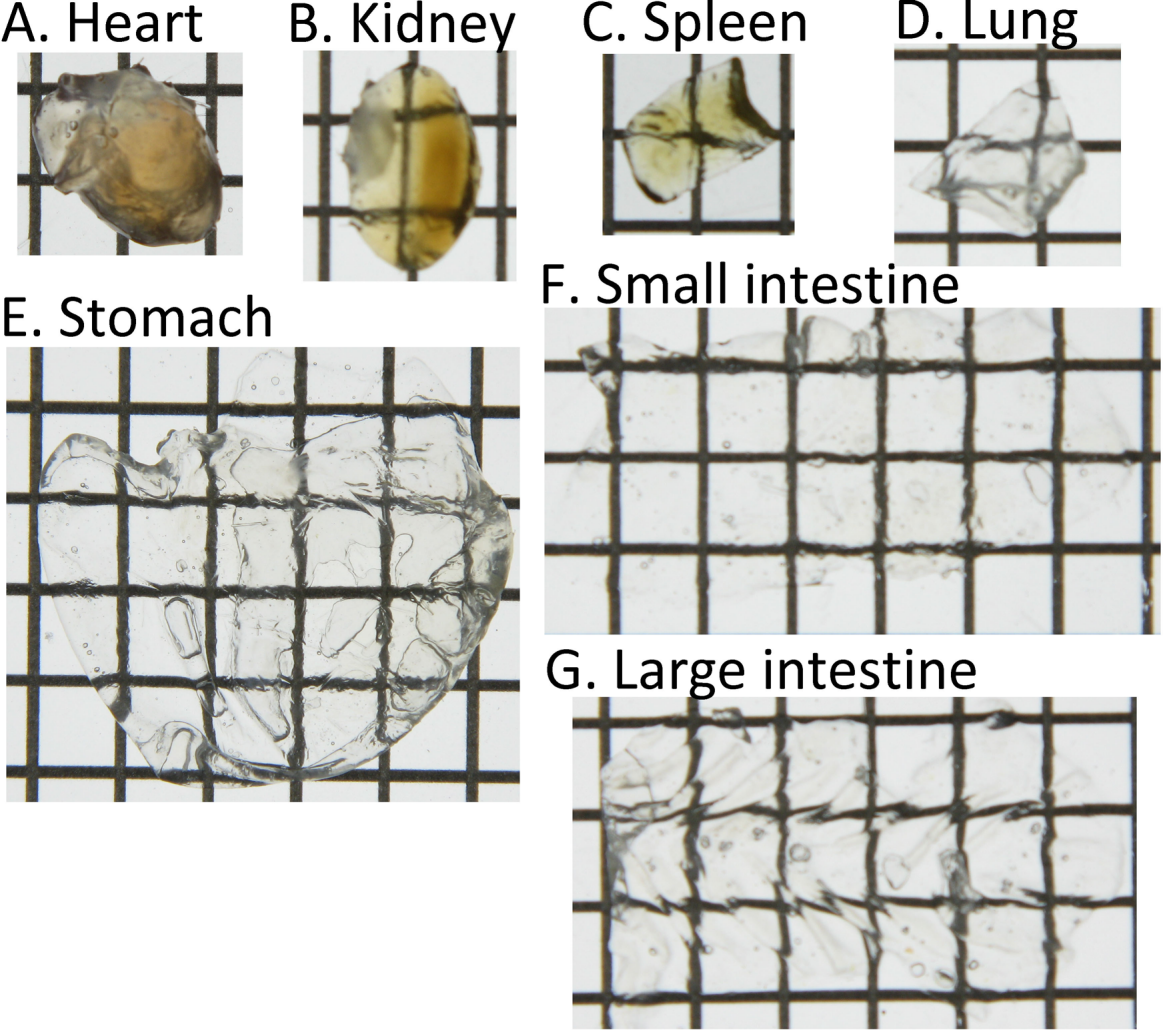
Transmission color images of each tissue treated with CUBIC. Each lattice indicates 4×4 mm.

**Fig 5:**
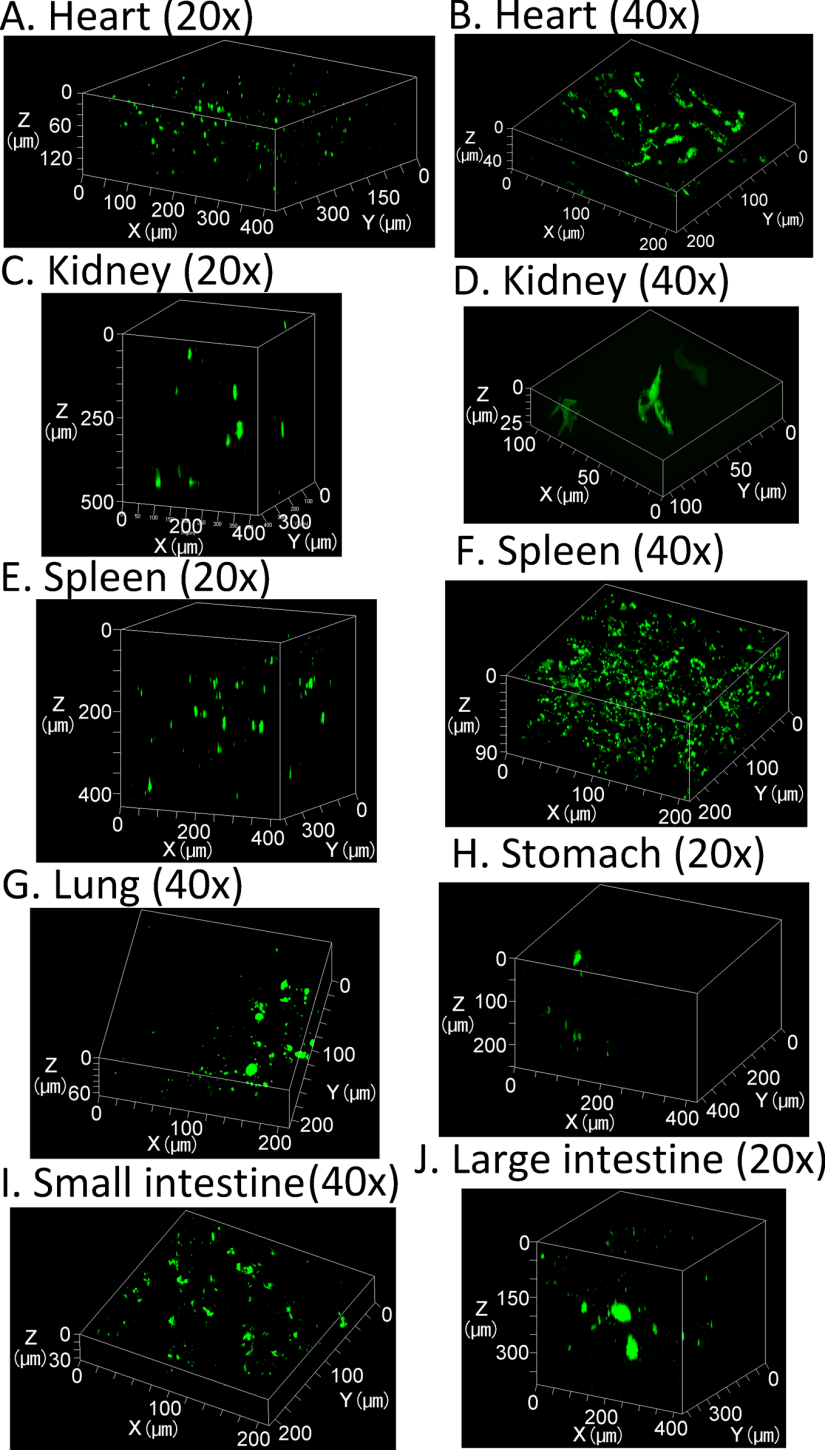
Observations of ZsGreen1 expression in various tissues treated with CUBIC. Three-dimensional maximum intensity projection. Acquisition conditions: lens, 20× dry (A, C, E, H, and J) or 40× oil-immersion (B, D, F, G, and I); laser, 488 nm; output, 5%; emission, 493–589 nm; master gain, (A) 951–1130, (B) 1081–1110, (C) 887–1152, (D) 732, (E) 823–977, (F) 732, (G) 1081–1110, (H) 1081–1110, (I) 1081–1110, and (J) 978–1146.

While the highest transgene expression is observed in the liver, about 4–6 orders of magnitude lower transgene expression can also be detected in other tissues including the heart, kidney, spleen, lung, stomach, and the small and large intestines (**Figure E in S1 File**). Thus, we evaluated transgene expression in other tissues to test the efficiency of CUBIC for three-dimensional observations of transgene expression. Transmitted images showed that other tissues also become transparent following CUBIC treatment (**Fig 4**). In particular, the stomach, and small and large intestines were almost colorless. The blood flow in these tissues is relatively low compared with that in the liver and kidney. Less blood flow would result in less erythrocytes in the tissues, which contribute to residual color. In terms of transgene expression, although the master gains for other tissues in CLSM were 1.7–2.2-fold higher than that for the liver, ZsGreen1 expression in other tissues was still detectable (**Fig 5**).

### Intracellular fate of plasmid DNA

**Fig 6:**
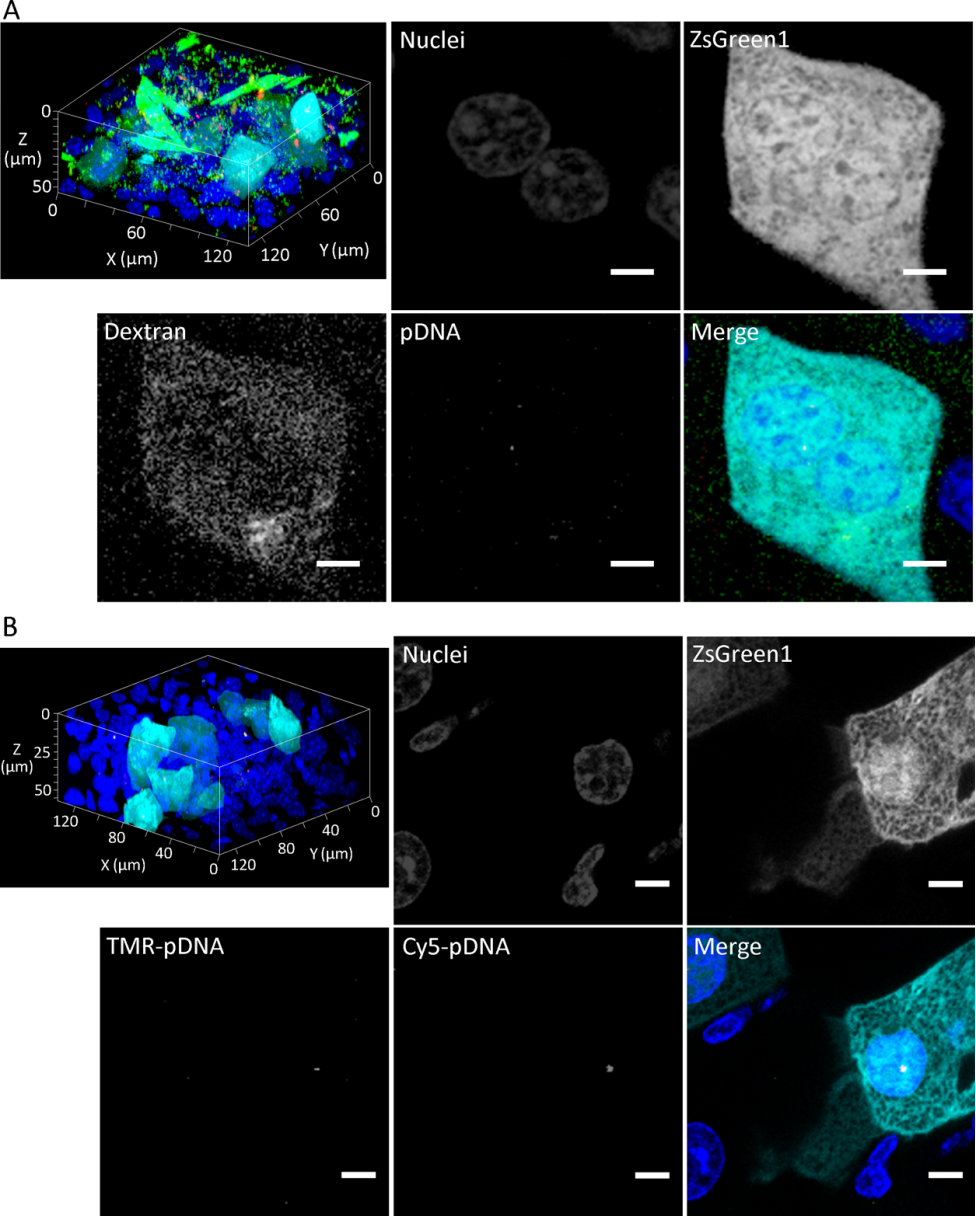
Intracellular fate of plasmid DNA in transgene expression-positive cells of the liver treated with CUBIC after hydrodynamic injection. Three-dimensional maximum intensity projections and enlarged planes. Scale bar: 5 μm. Nuclei (blue), ZsGreen1 (cyan), Cy5-plasmid DNA (red), (A) TMR-dextran (green), and (B) TMR-plasmid DNA (green). Acquisition conditions: (A) lens, 63× oil-immersion; laser, 405 nm (output, 12.5%; emission, 425–460 nm; master gain, 781–862), 488 nm (output, 0.5%; emission, 503–533 nm; master gain, 580), 543 nm (output, 3.0%; emission, 548–627 nm; master gain, 1153) and 633 nm (output, 4.0%; emission, 639–748 nm; master gain, 1150); (B) lens, 63× oil-immersion; laser, 405 nm (output, 12.5%; emission, 414–480 nm; master gain, 752–815), 488 nm (output, 0.4%; emission, 525–531 nm; master gain, 525–531), 543 nm (output, 5.0%; emission, 585–647 nm; master gain, 1150), and 633 nm (output, 4.0%; emission, 639–690 nm; master gain, 1132).

The intracellular fate of plasmid DNA, such as nuclear localization, is an important factor to determine transfection efficiency. Here, to test the capability of tissue clearing to reveal the intracellular fate of plasmid DNA, we delivered plasmid DNA to the liver via hydrodynamic injection and observed the cellular localization of plasmid DNA in transgene-positive cells of the liver. The macromolecular marker dextran was co-delivered with the plasmid DNA (**Fig 6A**). Punctate distribution of dextran might indicate fluid-phase endocytosis. However, both nuclear plasmid DNA and cytosolic dextran were localized in transgene-positive cells. Next, plasmid DNA encoding ZsGreen1, and TMR- and Cy5-labeled plasmid DNAs were co-delivered to the liver (**Fig 6B**). In transgene-positive cells, both TMR- and Cy5-labeled plasmid DNAs were co-localized in the nucleus, suggesting that several molecules of plasmid DNA were transferred together. This synchronized cellular distribution of plasmid DNA was consistent with the synchronized expression of the two plasmid DNAs encoding ZsGreen1 and tdTomato (**Fig 3**). In addition, we performed deep imaging of plasmid DNA (**Figure F in S1 File**). Observable depths of Cy5-labeled plasmid DNA were 62, 64 and 200 μm using Clear^T2^, SeeDB and CUBIC, respectively. Overall, CUBIC was the best tissue clearing method for multicolor deep imaging.

## Discussion

In this study, using tissue clearing methods, we achieved consecutive observations of transgene expression in the liver and other tissues without preparation of tissue sections. We compared tissue clearing methods to determine the optimal approach for detection of transgene expression. Among the tested methods, CUBIC was optimal for the observable depth. The maximum depth observed for ZsGreen1 was about 1.5 mm from the liver surface using CLSM with a 20× dry lens (NA: 0.5) (**Figs 1 and 3**). Although the transgene expression in other tissues was much lower than that in the liver, it was also possible to observe ZsGreen1 expression in other tissues (**Fig 5**). It is thought that the observable depth is dependent on the NA, magnification, WD, and transgene expression level. Certain approaches can be employed to improve the observable depth, such as using a higher performance lens and/or two-photon excitation microscopy. Importantly, CUBIC could determine the distribution of not only the transgene expression but also fluorescently labeled plasmid DNA. From this viewpoint, we succeeded in simultaneous observation of nuclei, transgene expression, the macromolecular marker dextran, and plasmid DNA in deep regions using the multicolor setting (**Fig 6 and Figure F in S1 File**).

Zhu et al. [9] have described the mechanisms of tissue clearing methods in detail. Reflective indices and light scattering in tissues are major factors. High light scattering due to lipids and extracellular matrices such as collagen fibers can be reduced by adjustment of reflective indices. Dehydration during tissue clearing is also an important factor. Among the tissue clearing methods, CUBIC [12, 13] uses a detergent, whereas Clear^T2^ [10] and SeeDB [11] do not. Thus, Clear^T2^ and SeeDB are compatible with blood vessel staining using carbocyanine dyes such as 1,1'-dioctadecyl-3,3,3'3'-tetramethylindocarbocyanine perchlorate. However, the performance of Clear^T2^ and SeeDB was insufficient, probably because of autofluorescence. Heme from residual erythrocytes in tissues largely contributes to color and autofluorescence. Aminoalcohols in CUBIC can remove heme from hemoglobin and successfully decolorize various tissues [13]. The decolorization efficiency of CUBIC might be different among tissues, mainly because of residual erythrocytes. Not only hemoglobin in erythrocytes but also other proteins including myoglobin and cytochrome P450 contain heme. In terms of the immersion time for tissue clearing, CUBIC requires a longer time than Clear^T2^ and SeeDB. Although heme could not be sufficiently removed at 1 day after immersion in CUBIC reagent 1, the transmission images indicated that CUBIC reagent 1 already cleared the liver more effectively than Clear^T2^ and SeeDB (**Figure A in S1 File**). In addition, prolonging the immersion times for Clear^T2^ and SeeDB had negligible effects on the observable depths **(Figure B in S1 File**). CLARITY can effectively clear the brain by removing lipids during electrophoresis [14]. However, specialized equipment is required for electrophoresis. Thus, we recommend CUBIC for observation of transgene expression in multiple tissues.

The observable depth was highly dependent on not only the tissue clearing method but also the microscope objective lens. A long distance type of high magnification lens enabled us to perform deep imaging of the intracellular fate of plasmid DNA. However, even using CUBIC, the observable depth of Cy5-labeled plasmid DNA (200 μm) was lower than that of ZsGreen1 and DAPI (at least 500 μm) (**Figure F in S1 File**). This result was probably due to the density of fluorochromes because signals from relatively large clusters of plasmid DNA were detectable at a depth of 500 μm from the surface. We considered that a depth of 200 μm would be sufficient to image the *in vivo* intracellular fate of plasmid DNA.

Among the types of fluorescent proteins, we tested two bright fluorescent proteins, ZsGreen1 and tdTomato. The relative quantum yields and extinction coefficients are 0.91 and 43,000 M^-1^cm^-1^ for ZsGreen1 and 0.69 and 138,000 M^-1^cm^-1^ for tdTomato, respectively. Thus, the brightness of tdTomato is 2.4-fold higher than that of ZsGreen1. However, in terms of the observable depth, ZsGreen1 was better than tdTomato. The half-lives of ZsGreen1 and tdTomato are sufficiently long [17, 18]. Thus, any differences would not be due to the amount of fluorescent proteins. The reason is still unclear, but major possibilities are laser output and mismatch in the excitation wavelength. One-hundred percent output of a 543 nm laser is equal to 3.3% output of a 488 nm laser. The excitation maximum of tdTomato (555 nm) is somewhat far from the laser excitation wavelength (543 nm), whereas that of ZsGreen1 (492 nm) is close to the laser excitation wavelength (488 nm). Photostability of ZsGreen1 was higher than that of tdTomato (**Figure C in S1 File**). In addition, fluorescent crosstalk of tdTomato in the green channel was not negligible (**Figure D in S1 File**). Taken together, ZsGreen1 might be more suitable for evaluating the spatial distribution of transgene expression than tdTomato, especially in multicolor settings.

It has been reported that positron emission tomography (PET) can be applied to imaging the spatial distribution of transgene expression [21]. Using PET, living subjects are observable. However, PET imaging and CLSM have resolutions at tissue and single cell levels, respectively. Thus, tissue clearing methods can determine the spatial distribution of transgene expression at the cellular level. In fact, cell-to-cell variability in transgene expression was obvious (**Figs 1, 3, 5 and 6**). Furthermore, the combination of tissue clearing methods and CLSM enable us to simultaneously determine multiple events such as transgene expression and the intracellular fate of plasmid DNA, whereas only one event (transgene expression) can be determined in PET imaging. For multiple signals, the combination of tissue clearing and CLSM can theoretically determine the type of transgene expression-positive cells by immunofluorescence.

Tissue clearing methods are compatible with mechanistic studies. Here, we analyzed the intracellular fate of plasmid DNA. Transgene expression-positive cells were merged with cytosolic dextran. It has been reported that hydroporation (i.e. a transient pore created by hydrodynamic injection) is a mechanism of high transfection efficiency [22]. Our results were consistent with the properties of hydroporation. A different distribution of cytosolic dextran indicated cell-to-cell variability in hydroporation, which might be one of the reasons for the cell-to-cell variability in transgene expression. Furthermore, nuclear membranes are thought to be barriers of transfection. As a mechanism of nuclear importation of plasmid DNA, it has been reported that transcription factors containing nuclear localization signals can deliver plasmid DNA to the nucleus [23–25].We found that several molecules of plasmid DNA were co-delivered to the nucleus (**Fig 6**), suggesting complex formation by several molecules of plasmid DNA with biological components such as cytosolic transcription factors and histones. In the case of liposomal vectors, the dissociation of the plasmid DNA from liposomes is a limiting step in transfection [26]. In hydrodynamic injection, naked plasmid DNA is more efficient than liposomal vectors, probably because dissociation is not needed for naked plasmid DNA.

## Conclusions

We applied tissue clearing methods to elucidate the spatial distribution of transgene expression and the intracellular fate of plasmid DNA in various murine tissues after hydrodynamic injection, including the liver, heart, kidney, spleen, lung, stomach, and the small and large intestines. Because more information can be obtained simultaneously without tissue sectioning by applying tissue clearing methods under *in vivo* conditions, we believe that this method would be useful for evaluation of gene delivery systems [27–33]. Therefore, three-dimensional and multi-color imaging methods for determining the spatial distribution of plasmid DNA and transgene expression in murine tissues may provide valuable information for the development of gene delivery systems.

## Supporting Information

S1 File Supporting figures. (DOCX)
