# Supplementary material for: Three-Dimensional Imaging of the Intracellular Fate of Plasmid DNA and Transgene Expression: ZsGreen1 and Tissue Clearing Method CUBIC Are an Optimal Combination for Multicolor Deep Imaging in Murine Tissues
Source: PLoS One. 2016 Jan 29;11(1):e0148233. doi: 10.1371/journal.pone.0148233 (PMC4732687; doi:10.1371/journal.pone.0148233)
Supplement: S1 File — (DOCX) [file pone.0148233.s001.docx]

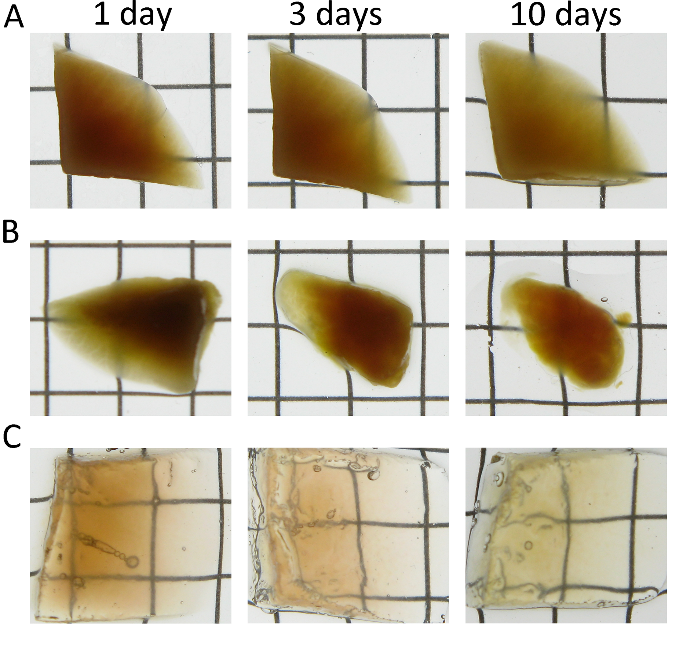


**Figure B. Observable depths using Clear^T2^ (A, C) and SeeDB (B, D) with prolonged immersion times.**

ZsGreen1 expression in the liver was observed at 14 days after immersion. (A, B) Depth coding. The color chart indicates depth on the Z-axis. (C, D) Maximum intensity projections (X–Z plane). Scale bar: 100 µm. Acquisition conditions: lens, 20× dry; laser, 488 nm; output, 5.0%, emission, 492–540 nm; master gain (A, C) 419–1130 and (B, D) 402–1150.


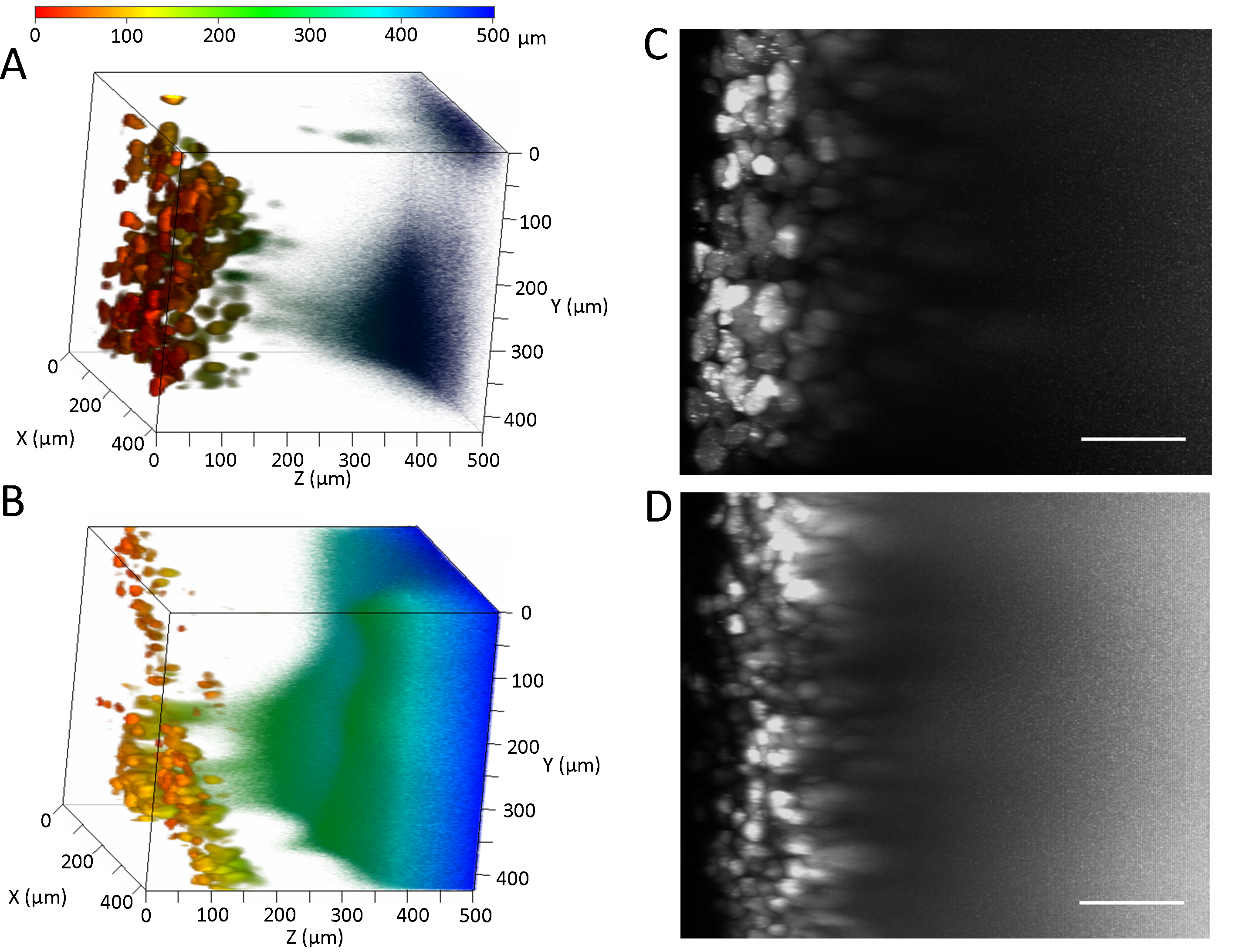


**Figure A. Time course of transmission color images of the liver after tissue clearing.**

(A) Clear^T2^, (B) SeeDB, and (C) CUBIC. Each lattice indicates 4×4 mm.


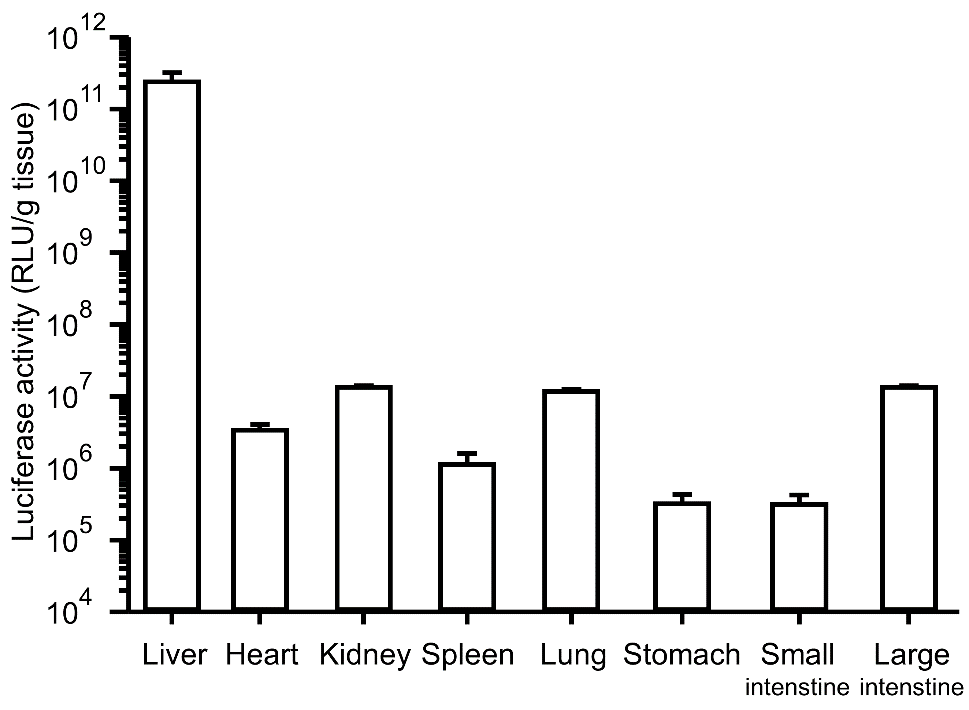


**Figure D. Fluorescent crosstalk of tdTomato emission in the green channel.**

The liver cleared by CUBIC was observed using a 40× oil-immersion lens after hydrodynamic injection of plasmid DNA ptdTomato-C1 into mice. (A) Green channel (488 nm laser; output, 6.5%; emission, 494 – 533 nm; master gain, 810). (B) Red channel (543 nm laser; output, 9%; emission, 550 – 670 nm; master gain, 713).

**Figure E. Comparison of transgene expression in various tissues after hydrodynamic injection of plasmid DNA encoding firefly luciferase.**

Data represent the mean + standard error of three experiments.


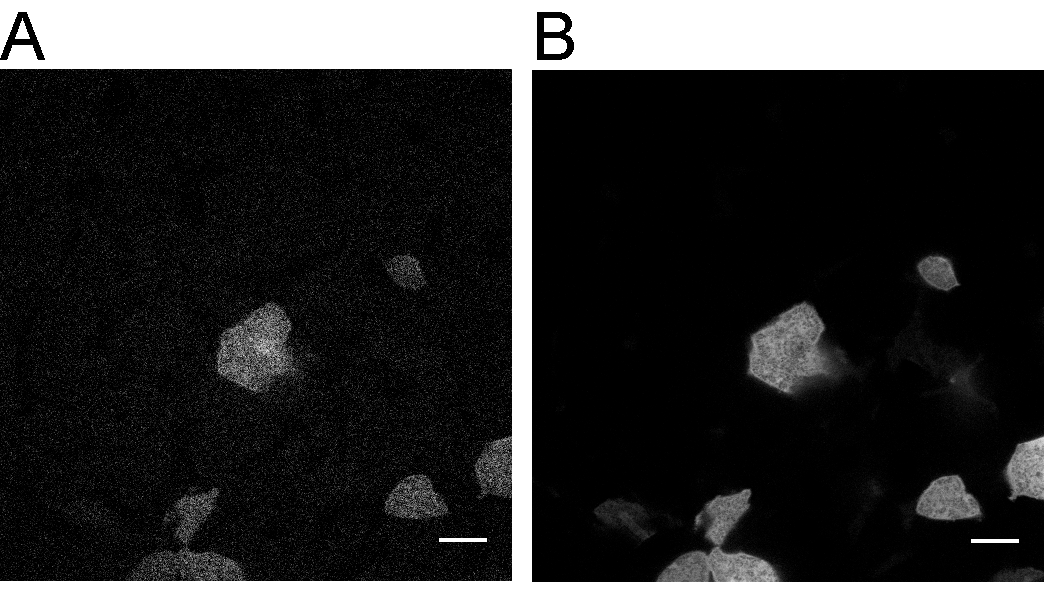


**Figure C. Comparison of photostabilities of ZsGreen1 and tdTomato.**

Declines in the fluorescence intensities of ZsGreen1 (emission 510 nm) and tdTomato (emission 570 nm) in PBS or CUBIC reagent 2 by consecutive irradiation with the excitation light (488 and 543 nm, respectively) were monitored using a fluorophotometer.


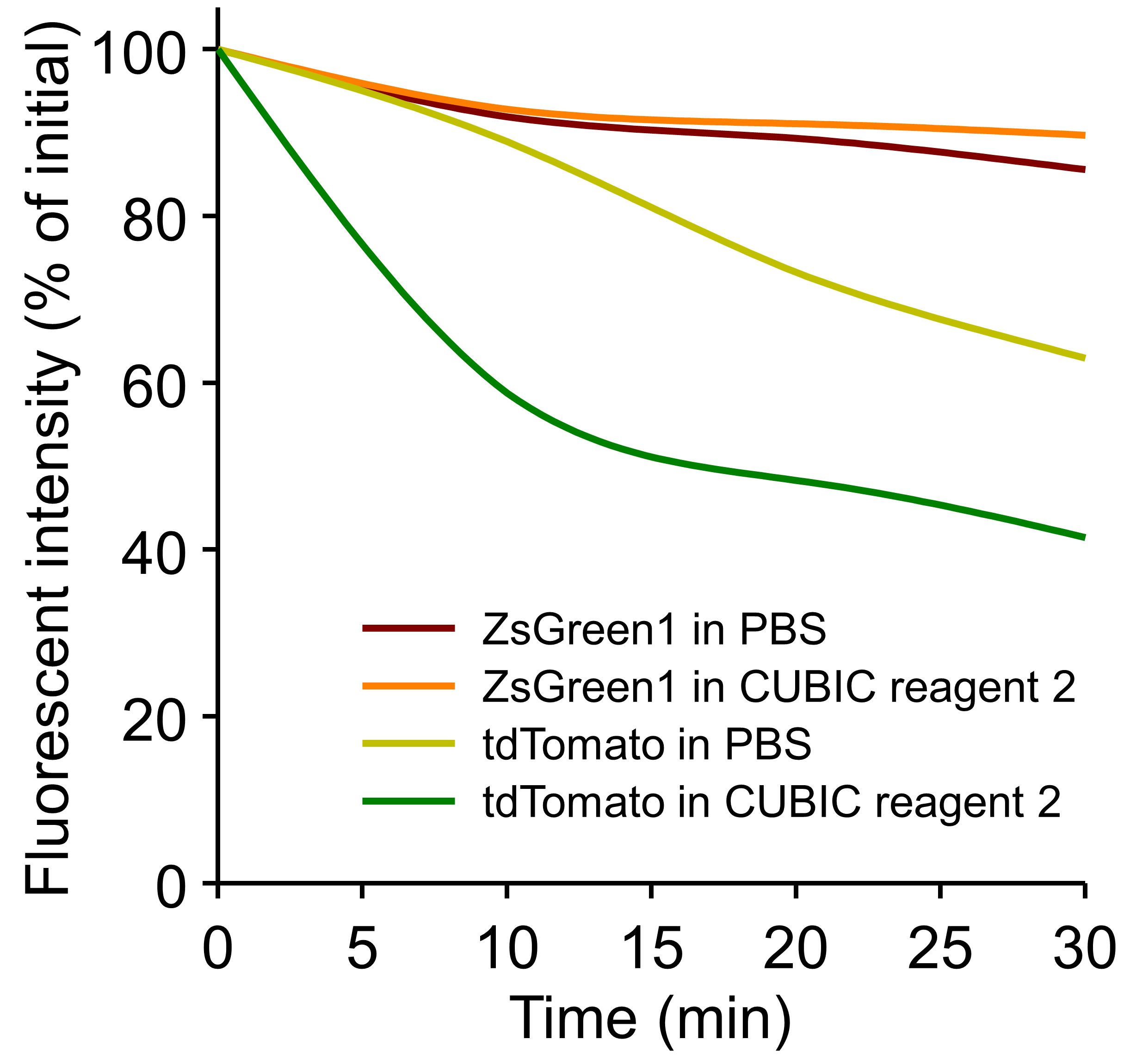

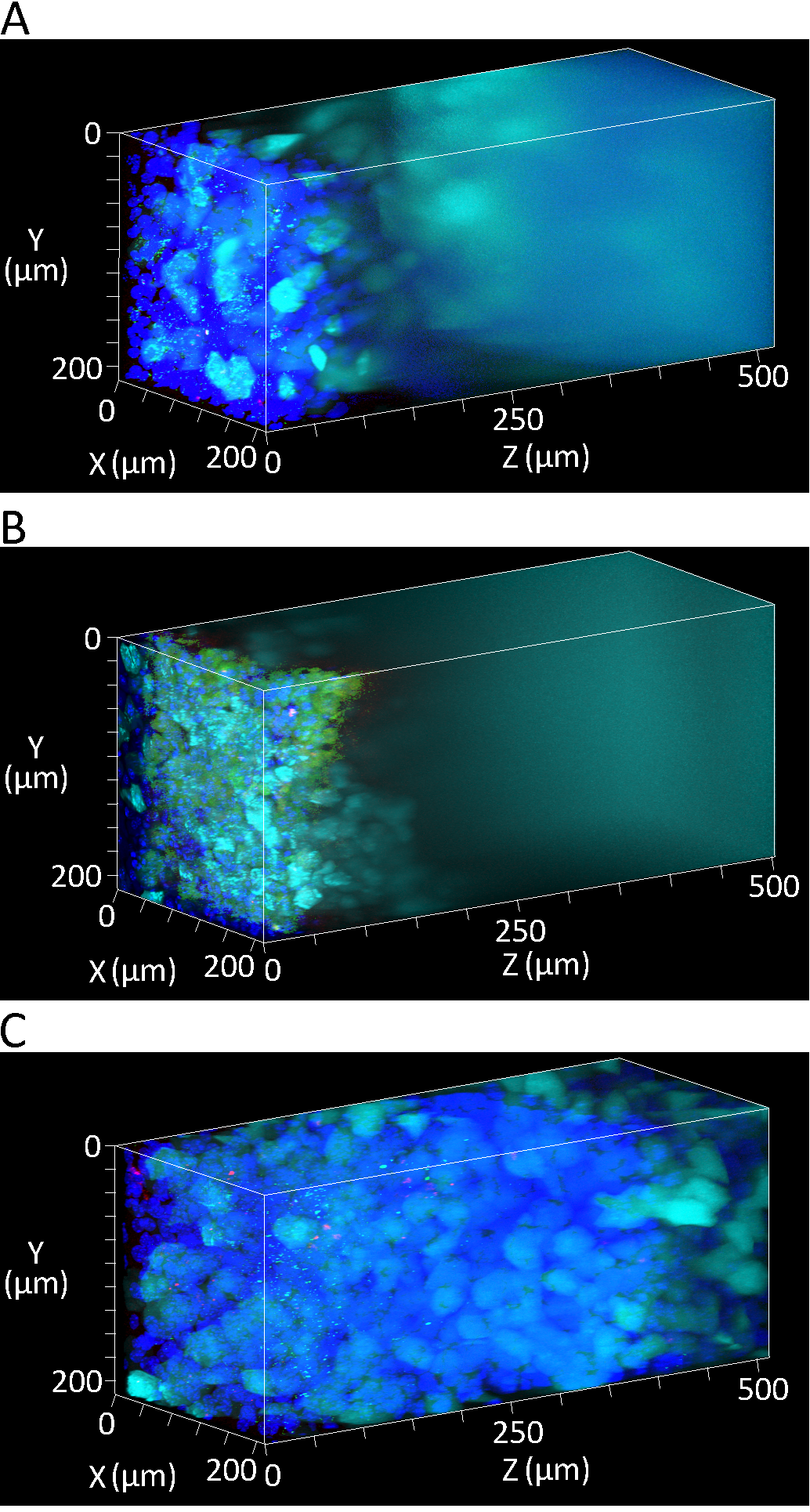


**Figure F. Comparison of multicolor deep imaging at high magnifications among tissue clearing methods.**

Three-dimensional maximum intensity projections using ClearT2 (A), SeeDB (B), and CUBIC (C). Nuclei (blue), ZsGreen1 (cyan), TMR-dextran (green), and Cy5-plasmid DNA (red). Acquisition conditions: lens, LD 40× water-immersion; (A) laser, 405 nm (output, 5.0%–20%; emission, 409–484 nm; master gain, 516–1200), 488 nm (output, 3.1%–10%; emission, 494–543 nm; master gain, 369–900), 543 nm (output, 5.0%–100%; emission, 553–631 nm; master gain, 759–900), and 633 nm (output, 5.0%–100%; emission, 637–755 nm; master gain, 1067–1200), (B) laser, 405 nm (output, 0.4%–5.0%; emission, 409–484 nm; master gain, 568–1118), 488 nm (output, 1.1%–5.0%; emission, 494–543 nm; master gain, 389–811), 543 nm (output, 5.0%–100%; emission, 553–631 nm; master gain, 758–900), and 633 nm (output, 7.2%–100%; emission, 637–755 nm; master gain, 957–1159), and (C) laser, 405 nm (output, 0.5%–0.7%; emission, 409–484 nm; master gain, 555–873), 488 nm (output, 1.0%–1.9%; emission, 494–543 nm; master gain, 495–566), 543 nm (output, 5.0%–35%; emission, 553–631 nm; master gain, 816–825), and 633 nm (output, 5.0%–19.3%; emission, 637–755 nm; master gain, 1078–1101).
